# Pregabalin and gabapentin for chronic low back pain without radiculopathy: a systematic review

**DOI:** 10.1055/s-0043-1764414

**Published:** 2023-06-28

**Authors:** Rafael Trindade Tatit, Arthur Werner Poetscher, Carlos Augusto Cardim de Oliveira

**Affiliations:** 1 Faculdade Israelita de Ciências da Saúde Albert Einstein, Departamento de Medicina, São Paulo SP, Brazil.; 2 Hospital Israelita Albert Einstein, Programa Locomotor, São Paulo SP, Brazil.; 3 Universidade da Região de Joinville, Departamento de Medicina, Joinville SC, Brazil.

**Keywords:** Low Back Pain, Back Pain, Chronic Pain, Pregabalin, Gabapentin, Systematic Review, Dor Lombar, Dor nas Costas, Dor Crônica, Pregabalina, Gabapentina, Revisão Sistemática

## Abstract

**Background** Chronic low back pain (CLBP) is a global health problem, and gabapentin and pregabalin are often used in the treatment of patients without associated radiculopathy or neuropathy. Therefore, determining their efficacy and safety is of enormous value.

**Objective** To examine the efficacy and safety of using gabapentin and pregabalin for CLBP without radiculopathy or neuropathy.

**Methods** We performed a search on the CENTRAL, MEDLINE, EMBASE, LILACS, and Web of Science data bases for clinical trials, cohorts, and case-control studies that evaluated patients with CLBP without radiculopathy or neuropathy for at least eight weeks. The data were extracted and inserted into a previously-prepared Microsoft Excel spreadsheet; the outcomes were evaluated using the Cochrane RoB 2 tool, and the quality of evidence, using the Grading of Recommendations Assessment, Development and Evaluation (GRADE) system.

**Results** Of the 2,230 articles identified, only 5 were included, totaling 242 participants. In them, pregabalin was slightly less efficacious than amitriptyline, the combination of tramadol/acetaminophen, and celecoxib, and pregabalin added to celecoxib showed no benefit when compared to celecoxib alone (very low evidence for all). On the other hand, although one study with gabapentin did not support its use in a general sample of patients with low back pain, another found a reduction in the pain scale and improved mobility (moderate evidence). No serious adverse events were observed in any of the studies.

**Conclusion** Quality information to support the use of pregabalin or gabapentin in the treatment of CLBP without radiculopathy or neuropathy is lacking, although results may suggest gabapentin as a viable option. More data is needed to fill this current gap in knowledge.

## Resumo

**Antecedentes** Dor lombar crônica (DLC) é um problema de saúde global, e a gabapentina e a pregabalina são frequentemente utilizadas no tratamento de pacientes sem radiculopatia ou neuropatia associada. Por isso, determinar sua eficácia e segurança é de enorme valor.

**Objetivo** Examinar a eficácia e segurança do uso de gabapentina e pregabalina no tratamento da DLC sem radiculopatia ou neuropatia.

**Métodos** Realizamos uma pesquisa nas bases de dados CENTRAL, MEDLINE, EMBASE, LILACS e Web of Science por ensaios clínicos, coortes e estudos de caso e controle que avaliassem pacientes com DLC sem radiculopatia ou neuropatia por pelo menos oito semanas. Os dados foram extraídos e inseridos em uma planilha previamente elaborada no programa Microsoft Excel; os desfechos foram avaliados com a ferramenta RoB 2 tool da Cochrane, e a qualidade das evidências, pelo sistema Grading of Recommendations Assessment, Development and Evaluation (GRADE).

**Resultados** Dos 2.230 artigos identificados, apenas 5 foram incluídos, com um total de 242 participantes. Neles, a pregabalina foi ligeiramente menos eficaz do que a amitriptilina, a combinação de tramadol/acetaminofeno, e o celecoxibe, assim como a pregabalina adicionada ao celecoxibe não mostrou benefício em comparação ao uso isolado de celecoxibe (evidência muito baixa para todos). Quanto à gabapentina, embora um estudo não respalde seu uso para uma amostra geral de pacientes com lombalgia, outro encontrou redução na escala de dor e melhora da mobilidade (evidência moderada). Nenhum evento adverso grave foi observado nos estudos.

**Conclusão** Há carência de informações de qualidade que sustentem o uso de pregabalina ou gabapentina no tratamento da DLC sem radiculopatia ou neuropatia, embora resultados possam sugerir que a gabapentina é uma opção viável. Mais dados são necessários para preencher essa atual lacuna no conhecimento.

## INTRODUCTION

Low back pain, typically defined as pain below the costal margin and above the inferior gluteal folds, with or without leg pain, 1 is usually classified according to duration as acute (< 6 weeks), subacute (6 to 12 weeks), or chronic (> 12 weeks). 2 Extremely common in populations throughout the world and occurring in all age groups, from children to the elderly, 3 4 5 it is a global health issue that has been responsible for 60.1 million disability-adjusted life years (DALYs) in 2015, 6 and it is currently the leading cause of disability. 7 It is estimated that up to 84% of all adults have at least 1 episode at some point in their lives, and it is one of the most common reasons for a primary care visit. 8 9 Although rapid improvement in pain and disability and return to work is the norm within the first month, 10 symptoms may persist beyond 12 weeks in some people. 11 When this happens, the use of medications to provide symptomatic pain relief, enabling the patient to participate in active therapies and encouraging increased function and improved coping can be implemented.

Furthermore, low back pain can be classified as mechanical, radicular (neuropathic), or primarily nociplastic in nature, 12 and the prevalence of the neuropathic pain ranges from 16% to 55% in patients with chronic low back pain (CLBP). 13 14 15 Therefore, drugs that were originally antiepileptics and their derivatives, mainly gabapentin and pregabalin, have been used as an alternative to other more traditionally recommended drugs in the treatment of CLBP – non-steroidal anti-inflammatory drugs (NSAIDs), duloxetine, tramadol, among others –, which have several limitations, adverse effects, and risks that are well-known with the long-term use. 16 17 18 19 20 21 However, evidence proving the real efficacy and safety of gabapentin and pregabalin in the treatment of CLBP, especially in the absence of radiculopathy or neuropathy, is still limited, with mixed and often inconclusive results. In addition, there are frequent reports of adverse effects associated with these medications, which highlights the need for further studies and analyses of the real pros and cons of their use. 22 23 24 25 Therefore, the present study aims to evaluate gabapentin and pregabalin in terms of their efficacy and safety in the treatment of CLBP without radiculopathy or neuropathy, according to the results published so far in the medical literature, through a systematic review.

## METHODS

### Eligibility criteria

Studies were considered eligible according to the following inclusion criteria: 1) randomized controlled clinical trials, cohort, and case-control studies; 2) participants aged 18 years or older, with CLBP or back pain without radiculopathy or neuropathy (we considered CLBP or back pain as pain for at least 2 months), without mixed conditions, that is, with no other painful complaints associated (such as low back pain and shoulder pain) unless the results were reported separately. There was no restriction regarding sex, place of birth/origin, or language of the publication.

Studies with pregnant women, with people in conditions eminently indicative of immediate surgical or interventional treatment, who had significant cognitive impairment, with low back or back pain caused by pathological entities (such as infections, neoplasms, metastases, osteoporosis, rheumatoid arthritis, fractures, or trauma) were excluded, as well as studies that were not published in full as articles (such as posters or conferences annals). In case of clinical trials, those whose protocols could not be found on international clinical trials databases were also excluded.

### Search methods

We identified studies through advanced searches on the Cochrane Central Register of Controlled Trials (CENTRAL), Medical Literature Analysis and Retrieval System Online (MEDLINE), Excerpta Medica Database (EMBASE), Latin American and Caribbean Health Sciences Literature (Literatura Latinoamericana y del Caribe en Ciencias de la Salud, LILACS, in Spanish), and Web of Science databases for articles published until August 20, 2022. More details of the search strategies are presented in Appendixes 1–5 ( Supplementary Material ). In addition, a manual search for eligible studies on the references of the publications found in the primary literature search was performed. Grey literature searches were not performed.

### Data collection and analysis

One reviewer extracted and gathered the search results and excluded clearly ineligible studies based on title and abstract. After that, the full articles of all remaining studies were retrieved. After reading these articles in full, those clearly ineligible were excluded. The remaining studies were reanalyzed by two reviewers, and only then were they excluded or included in the final composition of the review. We resolved any disagreements by consensus among the review authors. One of the reviewers manually extracted and inserted the data into a spreadsheet prepared by consensus by the reviewers using the Microsoft Excel (Microsoft Corp., Redmond, WA, United States) software.

One of the reviewers assessed the risk of bias of all included studies using version 2 of the Revised Cochrane risk-of-bias tool for randomized trials (RoB 2), 26 described in Appendix 6 ( Supplementary Material ). We classified each of the criteria as “low risk”, “some concerns”, and “high risk”. For the criteria classified as “some concerns”, we did not contact the trial authors for further information.

Efficacy data were examined according to previously-established outcome measures ( Appendix 7 - Supplementary Material ). Any serious adverse events were mentioned in separately from the less serious ones. As for the other outcomes, such as any other pain-related outcome indicating some improvement, they were assessed using the Descriptor Differential Scale (DDS) and a standard numerical rating scale (0 = “no pain”, 10 = “worst imaginable pain”). Adverse events were measured by the proportion of participants who experienced them.

We did not assess clinical heterogeneity for any of the clinical trials included, as they were very different from the start, both in terms of intervention and comparator, and in relation to the general population studied. Because of this too, only a meta-analysis of the proportion of adverse events experienced comparing gabapentin versus placebo between two studies could be carried out.

Among our outcomes, we used dichotomous data of known usefulness. 27 We would only perform a meta-analysis if there were at least two studies with sufficiently similar participants, interventions, comparisons, and measurement of outcomes. Otherwise, we would describe the results of comparable clinical trials in the review text. To assess and synthesize the quality of evidence for each result, we used the Grading of Recommendations Assessment, Development and Evaluation (GRADE) system, as recommended in the Cochrane Handbook for Systematic Reviews of Interventions, 28 based on the following domains: limitations of design, inconsistency of results, indirectness, imprecision, and other factors (such as publication bias). Finally, we developed “Summary of Findings” tables to present the certainty (or quality) of the body of evidence.

## RESULTS

### Description of the studies

**Figure 1:**
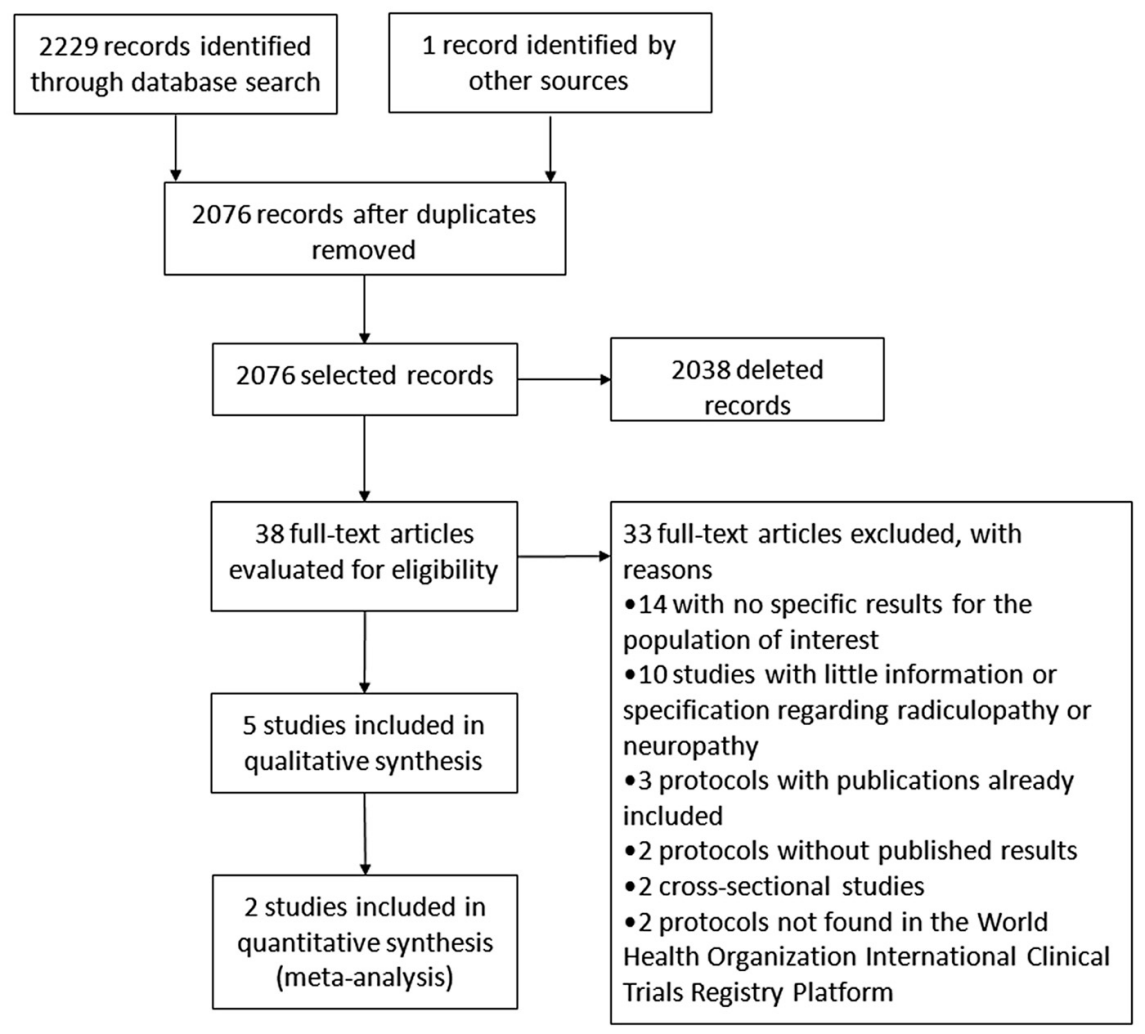
Flow diagram of the study.

We identified 2,230 potential articles through the primary electronic search and manual review of the research protocols found in it ( Figure 1 ). The main reasons for exclusion were because some articles did not contain a population of patients with low back pain without radiculopathy/neuropathy, not even after the division into groups (such as with and without radiculopathy/neuropathy), or they did not provide enough information about the presence of radiculopathy/neuropathy. Finally, less frequent were the study protocols in which there were no publications or dissemination of data until the end of our search, on August 20, 2022. All articles or protocols contained at least one alternative title in a language understandable to the authors. After the selection by title, all articles also contained at least one alternative abstract in an understandable language. Finally, all articles read in full were written in understandable language and, after a selection, five were included. The sample size of these studies ranged from 30 to 200 randomized participants, with a total of 445 participants.

However, because only one of the studies contained a pure sample of interest to us (McCleane, 2000 29 ), that is, the other studies also contained participants who did not fit our selection criteria (such as patients with radiculopathy or associated neuropathy), only a portion of the population of these other studies was included. In this case, the sample size of the five trials ranged from 20 to 93, with a total of 242 participants. All of these studies were randomized controlled clinical trials and had significant particularities, as can be seen in the Table 1 .

**Table 1:** Characteristics of the studies included in the present review

Study		McCleane, 2000 29	Atkinson et al., 2016 30	Kalita et al., 2014 31	Sakai et al., 2015 32	Romanò et al., 2009 33
**Methods**	**Design**	Double-blinded crossover RCT	Double-blinded RCT	Open, controlled RCT	Equivalence open CT	Crossover RCT, three intervention arms
	**Country**	N/A	United States	India	Japan	Italy
	**Language**	English	English	English	English	English
**Participants**	**Mean age in years**	42.4 (SD: ± 14.60)	56.04 (SD: ± 10.23)	41.5 (range: 21–65)	72.5 (range: 65–87)	53 ( ± SD: 16)
	**Sex: male – n (%)**	11 (45.83)	85 (78.7)	109 (54,5)	40 (66.67)	16 (44.44)
	**Total number**	30 randomized; 24 analyzed	108 randomized	200 randomized	65 randomized; 60 analyzed	42 randomized; 36 analyzed
	**Number considered for the present systematic review**	30 randomized; 24 analyzed	58 randomized (pain confined to the low back – Quebec I)	93 randomized (localized CLBP)	41 randomized; 38 analyzed (without neuropathic pain)	20 analyzed (unlikely neuropathic component – LANSS < 12)
	**Definition of CLBP**	Chronic benign nociceptive pain. Nociceptive pain: midline lumbar back pain with tenderness over a single interspinous ligament and with pain exacerbated by flexion of the back.	Pain ≤ T-6, secondary to degenerative disk or degenerative joint disease present “on a daily basis” for the previous 6 months or longer, of at least “moderate” intensity determined by DDS > 7.	Backache that lasts for more than 3 months which may be localized backache, associated with radiculopathy or with lumbar canal stenosis.	Neuropathic (Neuropathic Pain Screening Questionnaire ≥ 6) or nociceptive LBP that had been treated and followed up continuously for 3 months or longer.	LBP with symptoms for more than 6 months, due to disc prolapse, lumbar spondylosis, and/or spinal stenosis, with minimal VAS score at recruitment of 40 mm.
	**Inclusion criteria**	Adult patients with chronic benign nociceptive pain who consented to participate in the study. The model of nociceptive pain chosen was that of posttraumatic ligamentous low back pain, defined for the purposes of the study as midline low back pain with tenderness over a single interspinous ligament and with pain exacerbated by back flexion.	1) ages 21 to 70 years; 2) nonspecific low back pain primarily in the lumbar region, present on a daily basis for the previous 6 months or longer, adjectivally described as, of at least “mild” intensity (≥ 2 on a “0”to“10” numeric rating scale) and having an impact on 2 or more aspects of everyday life; 3) English-speaking, literate, able to understand the study, and communicate with the study team; 4) presently not a candidate for back surgery (1 prior back surgery permitted if it was less than 5 years ago); 5) agreement to discontinue muscle relaxants, antidepressants, and opioids at least 2 weeks before eligibility assessment and throughout the study (NSAIDs were permitted); and 6) if female, not pregnant or lactating, and with a negative pregnancy test at screening.	Consecutive patients with chronic low back pain (low back pain for more than 3 months) seen at the neurology service, who were between 18 and 75 years of age.	Patients with low back pain, aged 65 years or older, who had been treated and followed up continuously for 3 months or more at the orthopedic surgery outpatient clinic of the hospital in question.	Patients with chronic low back pain (symptom duration: more than 6 months, mean: 13 ± 6 months) due to disc prolapse, lumbar spondylosis, and/or spinal stenosis; minimum VAS score at recruitment: greater than 40 mm; age: over 18, under 75 years; having signed the consent form to participate in the study.
	**Exclusion criteria**	Patients with symptoms of neuropathic pain (shooting pain, paresthesia/ dysesthesia, allodynia or numbness), bony pain (pain arising from spinous process rather than interspinous ligament), with abnormalities on lumbar spine X-rays (either at the site of lumbar pain or at a level from which radiation or referral of pain to that area was possible), and patients who had previously undergone any surgical procedure for their back pain.	1) a major coexisting medical illness that might increase risks of gabapentin, or major surgical or nonsurgical intervention for any disorder within the past 12 months, because rehabilitation from treatment may confound study outcomes; 2) significant coexisting orthopedic or pain problems or back pain due to other disorders; 3) meeting DSM-IV criteria for alcohol or other substance use disorder; or current major depression or dysthymia; or lifetime diagnosis of bipolar disorder, psychosis or cognitive impairment disorder; 4) history of multiple adverse drug reactions or known allergy to gabapentin; 5) use of psychotropics, which would be continued during the study, or other drugs or agents which might interact with the study drug; 6) previous treatment with the study drug; 7) use of systemic corticosteroids or corticosteroid injections within 3 months of screening; or concurrent behavioral therapies, chiropractic treatment, or transcutaneous electrical nerve stimulation unit; 8) renal impairment; 9) hepatic impairment;10) hematologic abnormality; 11) pregnancy; and 12) use of experimental drugs or participation in other clinical trials within 2 months of screening for eligibility.	The patients with CLBA due to specific cause such as injury, infection, malignancy, collagen vascular disease, rheumatoid or seronegative arthritis, spinal tumors and vascular malformation were excluded. Patients on immunosuppression therapy, anticancer drugs, postorgan transplantation and postspinal surgery, CLBA patients below 15 years and above 65 years of age, those with pregnancy, lactation and severe neurological deficit due to radiculopathy or lumbar canal stenosis were excluded.	Patients who received other treatment for LBP, patients whose lower extremity symptoms were stronger than their low back pain, and patients with thoracolumbar compression fractures, tumorous infections of the spine, symptomatic lumbar disc herniation, spondylolisthesis ≥ grade II, and dementia.	Previous back surgery; diabetes; neurological disease; cardio-renal disease; history of gastric ulcers or intestinal bleeding; known allergy to the drugs under study; alcohol or drug abuse.
**Interventions**	**Intervention group**	Initial daily dose of 300 mg, increasing in 300 mg steps at weekly intervals to receive a total daily dose of 15 mg/kg/day gabapentin to the nearest 300 mg.	Gabapentin 300 mg orally 3 times a day up to a maximum of 1,200 mg orally 3 times a day for 12 weeks.	AMT 12.5 mg at bedtime for 2 weeks, followed by 25 mg for 4 weeks, then increased to 50 mg.	PG 75 mg before bedtime for 4 weeks.	Celecoxib (approximately 3–6 mg/kg/day) + placebo; PG (approximately 1 mg/kg/day the first week and then 2–4 mg/kg/day) + placebo; celecoxib (approximately 3–6 mg/kg/day) + PG (approximately 1 mg/kg/day the first week, and then 2–4 mg/kg/day). Each treatment lasted 4 weeks, with one week of discontinuation between treatments.
	**Control group**	Placebo with an increase in the number of capsules proportional to the equivalent increase in gabapentin capsules, for 6 weeks.	Inert placebo capsules identical in size and shape to the experimental capsules, one to three capsules taken orally three times daily for 12 weeks.	PG 75 mg twice daily for 2 weeks, followed by 150 mg twice daily for 4 weeks, then 300 mg twice daily.	TRAM/APAP twice-daily dosing (2 tablets per day; tramadol 75 mg and acetaminophen 650 mg per day) for 4 weeks.	
	**Follow-up (weeks)**	13 (6 weeks in each treatment period, followed by a 1-week washout phase).	12	14	4	15 (4 weeks in each treatment period with 1 week of washout between each one).
	**Specific drop-out rates**	Of the 30 patients originally recruited, 4 did not show up for review, one failed to complete the study record sheets, and one withdrew early due to gabapentin side effects.	Of the patients who dropped out before completing 12 weeks, 19 had been assigned to gabapentin and 17, to placebo. The main reason for discontinuation was similar in both groups and, in most cases, it was due to adverse effects or lack of efficacy; a considerable proportion were lost to follow-up, and no reason could be obtained for withdrawal.	15 patients in each study arm were lost to follow-up for unknown reasons; 11 patients in the AMT group and 12 in the PG group discontinued the intervention because of side effects.	2 patients in the PG group and 3 in the TRAM/APAP group discontinued the intervention due to side effects. No patient was lost to follow-up during the study.	4 out of 42 recruited patients discontinued treatment within the first 2 weeks due to adverse events (epigastralgia and/or nausea), 1 taking PG monotherapy, 1 taking celecoxib monotherapy, and 2 taking PG + celecoxib. Of the 38 remaining participants, another 2 also discontinued treatment (1 for logistical reasons at work, the other, for trauma due to a car accident).
**Outcomes**	**Primary**	Analgesic efficacy (mean pain score of patients and controls, on a scale of 0 to 10), mobility (mean mobility score – ability to flex the back, on a scale of 0 to 10, self-reported by the patient), analgesic consumption and patient preference for study medication.	Transformed DDS-pain intensity scores adjusted for time (time frame: baseline to week 12 with interim measurements at weeks 1, 2, 3, 4, 5, 7, and 9).	Mean pain reduction (VAS score reduction > 50%) after 14 weeks.	Pain, assessed using the VAS (scale from 0 to 10) and activities of daily living, assessed using the RMDQ, Short-Form McGill Pain Questionnaire, EuroQol quality-of-life scale, and Geriatric Depression Scale.	Mean pain reduction after different treatment regimens by the VAS (100 mm).
	**Secondary**		RMDQ adjusted for time (time frame: baseline to week 12 with interim measurements at weeks 1, 2, 3, 4, 5, 7, and 9).	Improvement in disability > 20% and improvement of adverse effects after 14 weeks.		Adverse effects due to the treatments.
**Results considered for the present systematic review**	**Efficacy**	Gabapentin: mean pain scale dropped from 7.10 (95%CI: 6.26–7.94) to 6.39 (95%CI: 5.39–7.39) ( *p * < 0.05). Placebo: no significant decrease in mean pain, from 7.52 (1.94) to 7.13 (2.34). Gabapentin: mean mobility increased from 4.65 (95%CI: 3.84–5.46) to 5.46 (95%CI: 4.50–6.42) ( *p * < 0.01). Placebo: non-significant decrease in mean mobility, from 5.07 (2.08) to 5.05 (2.04).	ITT: decrease in DDS both in the gabapentin and in the placebo groups ( *p* < 0.0001), with a reduction of about 30% on DDS and no difference between the interventions ( *p* = 0.423). Per-protocol analysis: reduction in pain using a standard verbal numerical rating scale from 0 to 10 in both groups (5.8 to 3.5 and 5.7 to 4.1; *p * < 0.0001), with no significant difference between the groups (2.2 versus 1.6; *p* = 0.253).	ITT: for the outcome reduction of pain intensity in the VAS reported by the participant of 50% or more, we obtained values of 32.6% (15 of 46) and 53.2% (25 of 47) (0.61 [95%CI: 0.37–1.01]; *p * = 0.05) for the PG and AMT groups respectively. As for the functionality analysis, ODI reduction greater than 20%, we obtained values of 39.1% (18 of 46) and 65.96% (31 of 47) (0.59 [95%CI: 0.39–0.90]; *p * = 0.01) for the PG and AMT groups respectively.	Pain improvement in the VAS (scale from 0 to 10), there was significant improvement after 4 weeks in both groups ( *p* < 0.05 for both); however, in the TRAM/APAP group, this improvement could already be observed after 2 weeks ( *p * < 0.05). As for the functional improvement by the RMDQ, no significant improvement was noted for the PG group, whereas for the TRAM/APAP group, a significant improvement was observed after 2 weeks of administration.	PG versus celecoxib: only celecoxib has been shown to improve pain score by VAS (43.8 [SD: ± 12.9] to 32.5 [SD: ± 15.5]; *p * = 0.01). PG + celecoxib versus PG: greater decrease in pain with the combined use of drugs (45.1 [SD: ± 14.2] to 32.9 [SD: ± 13.9] versus 49.4 [SD: ± 13.2] to 50.7 [SD: ± 13.8]; *p* = 0.0002). PG + celecoxib versus celecoxib: no superiority of the combined regimen compared to monotherapy in reducing pain (45.1 [SD: ± 14.2] to 32.9 [SD: ± 13.9] versus 43.8 [SD: ± 12.9] to 32.5 [SD: ± 15.5]; *p* = 0.9).
	**Safety**	Adverse events were reported in both groups, with a significantly higher number during the gabapentin use (9 out of 30), versus placebo (2 out of 30) (4.5 [95%CI: 1.06–19.11]; *p* = 0.04).	At least 1 adverse event: gabapentin – 49 out of 55 (89%) versus placebo – 35 out 53 (66%); *p* = 0.008. At least 1 moderate to severe adverse event: gabapentin – 30 out of 55 (55%) versus placebo – 17 out of 53 (32%); *p * = 0.03. No serious adverse events were reported.	Adverse events were reported in both groups, with no significant differences between them (18 patients in the AMT and 21 in the PG group; *p* = 0.48).	Adverse events were reported in both groups, with a significantly higher number in the group that was given TRAM/APAP (21 out of 33), versus PG (12 out of 32) (0,59 [95%CI: 0.35-0.99]; *p* = 0.04).	4 patients out of 42 recruited discontinued treatment due to adverse events (epigastralgia and/or nausea): 1 taking PG; 1 taking celecoxib; 2 taking PG + celecoxib.

Abbreviations: AMT, amitriptyline; CI, confidence interval; CLBA, chronic low backache; CLBP, chronic low back pain; CT, clinical trial; DDS, Descriptor Differential Scale; DSM-IV, Diagnostic and Statistical Manual of Mental Disorders - IV; ITT, intention-to-treat analysis; LANSS, Leeds Assessment of Neuropathic Symptoms and Signs; LBP, low back pain; N/A, not available; NSAIDs, nonsteroidal anti-inflammatory drugs; ODI, Oswestry Disability Index; PG, pregabalin; RCT, randomized clinical trial; RMDQ, Roland Morris Disability Questionnaire; SD, standard deviation; TRAM/APAP, tramadol/acetaminophen combination tablets; VAS, Visual Analogue Scale.

Among the outcomes previously established for the present research ( Appendix 7 Supplementary Material ), only those described as follows were reported in the selected studies and were applied to the present review. The primary outcome, participant-reported reduction in pain intensity of 50% or more, was measured through the Visual Analog Scale (VAS). The assessment of functional improvement was measured through the Oswestry Disability Index (ODI) and Roland Morris Disability Questionnaire (RMDQ).

### Risk of bias in the studies included

**Figure 2:**
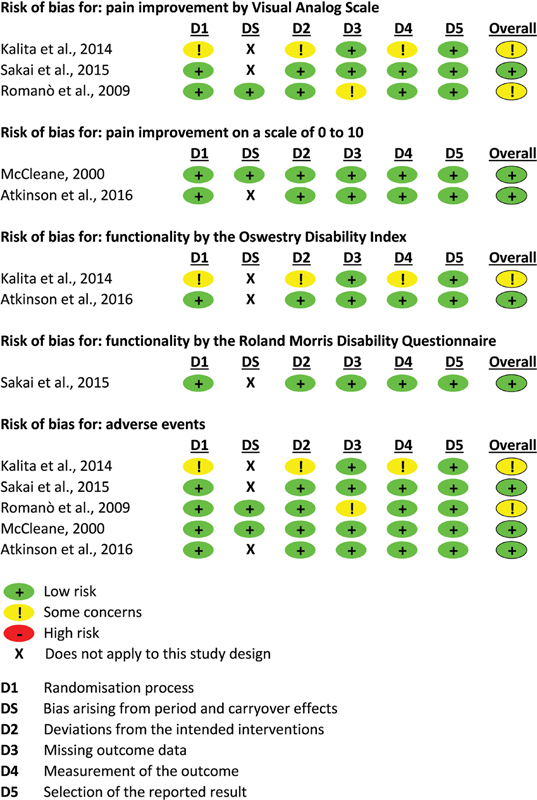
Assessment of the risk of bias according to each outcome presented.

The assessment of the risk of bias is presented in Figure 2 . Three of the five studies were considered to have a low risk of bias: (McCleane, 2000; 29 Sakai et al., 2015; 32 and Atkinson et al., 2016. 30

In terms of ‘allocation’, the five studies selected 29 30 31 32 33 reported a randomization procedure. Of these, one 31 did not provide clear information on allocation sequence concealment, two 29 30 adequately described treatment allocation concealment, and two 32 33 were not explicit in the description of treatment allocation concealment, although we can infer that there was. In the five studies, any baseline differences observed among the intervention groups appear to be by chance.

Two 29 30 of the studies reported blinding of the patients, caregivers, and outcome assessors, and two 32 33 reported blinding of the outcome evaluators. The latter 33 presented an incongruity when stating in the introduction that it was a single-blinded study, while in the methods section the authors 33 stated that it was a double-blinded study. One of the studies 31 did not blind the patients, caregivers or outcome assessors.

In terms of incomplete outcome data, for the continuous outcomes, with the availability of data from 95% of the participants, the dropout rate was considered small. For the dichotomous results, the dropout rate was considered small when data from at least 80% of the participants were available. When the dropouts were justified, such as in case of adverse events, with the description of the group they were in, we considered those studies less prone to bias. Three of the studies 29 32 33 reported small dropout rates; the other two studies 30 31 reported dropout rates higher than 20%, but only these two studies performed an intention-to-treat analysis (ITT).

The studies showed differences in baseline characteristics and time of outcome assessment. The mean age was much higher in the study by Sakai et al., 32 (72.5 years) than in the others (41.5 to 56.04 years), and the proportion of male patients was higher in Atkinson et al. 30 (78.7% versus 44.44% to 66.67%). Furthermore, the follow-up varied from 4 32 33 to 14 31 weeks. On the other hand, all studies at least avoided cointerventions (or advised that they should only be performed when necessary, not regularly). In one study, 30 maintenance of stable complementary NSAID therapy was allowed. In another study, 29 the patients were allowed to remain on a stable dose of NSAIDs and to continue the use of a compound analgesic based on paracetamol and codeine as rescue analgesia. However, they were asked to remain using the same compound preparation and to take it only when needed, not regularly.

None of the studies showed significant conflicts of interest. We did not create funnel plots to assess potential publication biases due to the small number of studies included.

### Effects of interventions

See “Summary of Findings” ( Tables 2 , 3 , 4 and 5 ).

**Table 2:** Summary of findings

Gabapentin compared to placebo for nociceptive chronic low back pain			
Patient or population: chronic nociceptive pain (midline low back pain with tenderness over a single interspinous ligament and with pain exacerbated by back flexion).			
Outcomes	Results	Subgroup analyzed (total population)	Certainty of the evidence (GRADE)
Change in self-reported pain by patients using a scale from 0 to 10 between the first and sixth weeks of treatment (per-protocol analysis)	During the gabapentin use, the mean pain scale dropped from 7.10 (95%CI: 6.26–7.94) to 6.39 (95%CI: 5.39–7.39) ( *p * < 0.05). During placebo use, there was no significant decrease in mean pain, from 7.52 (1.94) to 7.13 (2.34)	24 (30) 29	⊕⊕⊕⊝ MODERATE ^a^
Change in mean mobility self-reported by patients between the first and sixth weeks of treatment (per-protocol analysis)	During the gabapentin use, mean mobility increased from 4.65 (95%CI: 3.84–5.46) to 5.46 (95%CI: 4.50–6.42) ( *p* < 0.01). During placebo use, there was a non-significant decrease in mean mobility, from 5.07 (2.08) to 5.05 (2.04)	24 (30) 29	⊕⊕⊕⊝ MODERATE ^a^

Abbreviations: CI, confidence interval; GRADE, Grading of Recommendations Assessment, Development and Evaluation.

Notes: GRADE Working Group grades of evidence: high certainty – we are very confident that the true effect lies close to that of the estimate of the effect; moderate certainty – we are moderately confident in the effect estimate: the true effect is likely to be close to the estimate of the effect, but there is a possibility that it is substantially different; low certainty – our confidence in the effect estimate is limited: the true effect may be substantially different from the estimate of the effect; very low certainty – we have very little confidence in the effect estimate: the true effect is likely to be substantially different from the estimate of effect. ^a^ Lowered a level because of severe inaccuracy due to very few events in the study.

**Table 3:** Summary of findings

Pregabalin compared to amitriptyline in the group of patients with chronic low back pain			
Patient or population: patients with chronic low back pain (pain for more than 3 months) without specific cause and significant neurological deficit			
Outcomes	Results	Subgroup analyzed (total population)	Certainty of the evidence (GRADE)
Pain relief (≥ 50% improvement in VAS score) at 14 weeks (ITT)	For the outcome reduction of pain intensity in the VAS reported by the participant of 50% or more, we obtained values of 32.6% (15 of 46) and 53.2% (25 of 47) (0.61 [95%CI: 0.37–1.01]; *p* = 0.05) for the PG and AMT groups respectively.	93 (200) 31	⊕⊝⊝⊝ VERY LOW ^a, b, c^
Reduction in ODI (version 2) (> 20%) in 14 weeks (ITT)	As for the functionality analysis, ODI reduction greater than 20%, we obtained values of 39.1% (18 of 46) and 65.96% (31 of 47) (0.59 [95%CI: 0.39–0.90]; *p* = 0.01) for the PG and AMT groups respectively.	93 (200) 31	⊕⊝⊝⊝ VERY LOW ^a, b, c^

Abbreviations: AMT, amitriptyline; CI, confidence interval; GRADE, Grading of Recommendations Assessment, Development and Evaluation; ITT, intention-to-treat analysis; ODI, Oswestry Disability Index; PG, pregabalin; VAS, Visual Analogue Scale.

Notes: GRADE Working Group grades of evidence: high certainty – we are very confident that the true effect lies close to that of the estimate of the effect; moderate certainty – we are moderately confident in the effect estimate: the true effect is likely to be close to the estimate of the effect, but there is a possibility that it is substantially different; low certainty – our confidence in the effect estimate is limited: the true effect may be substantially different from the estimate of the effect; very low certainty – we have very little confidence in the effect estimate: the true effect is likely to be substantially different from the estimate of effect. ^a^ Lowered two levels because of very serious limitations of the study, due to some concerns about the risk of bias in the study by not blinding the allocation; ^b^ lowered a level due to severe inaccuracy, because the sample size criterion was not met in the per-protocol analysis; ^c^ lowered a level due to “Indirectness” of the severe evidence, as only part of the population was of interest to us.

**Table 4:** Summary of findings

Pregabalin compared to tramadol/acetaminophen in the group of patients with chronic low back pain without neuropathy			
Patient or population: patients aged 65 years or older who had chronic low back pain			
Outcomes	Results	Subgroup analyzed (total population)	Certainty of the evidence (GRADE)
Improvement of pain in the VAS (scale from 0 to 10) after 2 weeks (per-protocol analysis)	There was a significant improvement in pain ( *p* < 0.05) only in the group that took TRAM/APAP	38 (65) 32	⊕⊝⊝⊝ VERY LOW ^a, b, c^
Improvement of pain in the VAS (scale from 0 to 10) after 4 weeks (per-protocol analysis)	There was a significant improvement in pain ( *p* < 0.05) in both the PG and TRAM/APAP groups	38 (65) 32	⊕⊝⊝⊝ VERY LOW ^a, b, c^
Functional improvement by RMDQ after 2 weeks (per-protocol analysis)	There was a significant functional improvement ( *p* < 0.05) only in the group that took TRAM/APAP	38 (65) 32	⊕⊝⊝⊝ VERY LOW ^a, b, c^
Functional improvement by RMDQ after 4 weeks (per-protocol analysis)	There was a significant functional improvement ( *p* < 0.05) only in the group that took TRAM/APAP	38 (65) 32	⊕⊝⊝⊝ VERY LOW ^a, b, c^
Efficacy of each agent rated as “remarkably effective”, “effective” and “ineffective” (per-protocol analysis)	Greater efficacy with the use of TRAM/APAP, compared to PG ( *p* < 0.05)	38 (65) 32	⊕⊝⊝⊝ VERY LOW ^a, b, c^

Abbreviations: GRADE, Grading of Recommendations Assessment, Development and Evaluation; PG, pregabalin; RMDQ, Roland Morris Disability Questionnaire; TRAM/APAP, tramadol/acetaminophen combination tablets; VAS, Visual Analogue Scale.

Notes: GRADE Working Group grades of evidence: high certainty – we are very confident that the true effect lies close to that of the estimate of the effect; moderate certainty – we are moderately confident in the effect estimate: the true effect is likely to be close to the estimate of the effect, but there is a possibility that it is substantially different; low certainty – our confidence in the effect estimate is limited: the true effect may be substantially different from the estimate of the effect; very low certainty – we have very little confidence in the effect estimate: the true effect is likely to be substantially different from the estimate of effect. ^a^ Lowered a level because of severe study limitations due to some concerns about the risk of bias from non-blinding of patients; ^b^ lowered a level due to severe inaccuracy, because the sample size criterion was not met in the per-protocol analysis; ^c^ lowered a level due to “indirectness” of the severe evidence, as only part of the population was of interest to us.

**Table 5:** Summary of findings

Pregabalin compared to celecoxib in the group of patients with unlikely neuropathic component (LANSS < 12)			
Patient or population: patients with chronic low back pain due to disc prolapse, lumbar spondylosis and/or spinal stenosis, with an unlikely neuropathic component (LANSS < 12)			
Outcomes	Results	Subgroup analyzed (total population)	Certainty of the evidence (GRADE)
Improvement in VAS pain after 4 weeks of treatment (per-protocol analysis)	Only in the celecoxib group was there a significant decrease in the pain score (mean self-reported VAS from 43.8 ± 12.9 to 32.5 ± 15.5; *p* = 0.01)	20 (42) 33	⊕⊝⊝⊝ VERY LOW ^a, b, c^
**Pregabalin and celecoxib compared with celecoxib monotherapy in the group of patients with unlikely neuropathic component (LANSS < 12)**			
**Patient or population:** **patients with chronic low back pain due to disc prolapse, lumbar spondylosis and/or spinal stenosis, with an unlikely neuropathic component (LANSS < 12)**			
**Outcomes**	**Results**	**Subgroup analyzed (total population)**	**Certainty of the evidence (GRADE)**
				Improvement in VAS pain after 4 weeks of treatment (per-protocol analysis)	There was no superiority of the combined regimen compared to monotherapy ( *p* = 0.9)	20 (42) 33	⊕⊝⊝⊝ VERY LOW ^a, b, c^

Abbreviations: GRADE, Grading of Recommendations Assessment, Development and Evaluation; LANSS, Leeds Assessment of Neuropathic Symptoms and Signs; VAS, Visual Analogue Scale.

Notes: GRADE Working Group grades of evidence: high certainty – we are very confident that the true effect lies close to that of the estimate of the effect; moderate certainty – we are moderately confident in the effect estimate: the true effect is likely to be close to the estimate of the effect, but there is a possibility that it is substantially different; low certainty – our confidence in the effect estimate is limited: the true effect may be substantially different from the estimate of the effect; very low certainty – we have very little confidence in the effect estimate: the true effect is likely to be substantially different from the estimate of effect. ^a^ lowered a level because of severe study limitations due to some concerns about risk of blinding bias; ^b^ lowered a level because of severe inaccuracy due to very few events in the study; ^c^ lowered a level due to “indirectness” of the severe evidence, as only part of the population was of interest to us.

### Gabapentin compared to placebo

The study by McCleane, 29 the only one that contained a pure sample of participants of our interest, obtained a reduction in the mean pain 0-10 verbal numeric rating scale only during the use of gabapentin: from 7.10 (95% confidence interval [95%CI]: 6.26–7.94) to 6.39 (95%CI: 5.39–7.39); *p* < 0.05–moderate degree of evidence (GRADE). One of the participants withdrew due to the side effects of gabapentin, and adverse events were reported in both groups, however, with a significant higher number among those using gabapentin (9 out of 30) than among those taking placebo (2 out of 30) (risk ratio: 4.5 [95%CI: 1.06–19.11]; *p* = 0.04). On the other hand, Atkinson et al. 30 obtained, in the ITT analysis, a decrease in DDS both in the gabapentin and in the placebo groups ( *p* < 0.0001), with a reduction of about 30% in DDS and no difference between the interventions ( *p* = 0.423). In the exploratory per-protocol analysis using a 0-10 verbal numeric rating scale, there was also a reduction in pain in both groups (from 5.8 to 3.5 and from 5.7 to 4.1; *p* < 0.0001), with no significant difference between them (2.2 versus 1.6; *p* = 0.253). Importantly, these results from Atkinson et al. 30 referred to a mixed population of patients with radiating (46 patients) and non-irradiated (62 patients) pain, but the reduction in intensity was similar among these participants, both within and between treatment arms (none of the *p* -values from the mixed-model analysis was significant). Furthermore, a greater proportion of individuals in the gabapentin group reported at least 1 adverse event (49 out of 55 [89%] versus 35 out of 53 [66%]; *p* = 0.008) or experienced at least 1 moderate to severe adverse event (30 out of 55 [55%] versus 17 out of 53 [32%]; *p* = 0.03), but no serious adverse events were reported.

**Figure 3:**
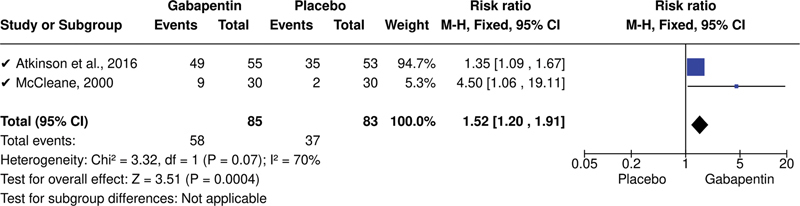
Forest plot of comparison: gabapentin versus placebo, proportion of adverse events experienced. Abbreviation: CI, confidence interval.

As would be expected, the meta-analysis results showed that both studies 29 30 had a greater presence of adverse events with the use of gabapentin when compared to placebo ( Figure 3 ) (risk ratio: 1.52 [95%CI: 1.20–1.91]; *p* = 0.0004).

### Pregabalin compared to amitriptyline

For the subgroup of patients with localized CLBP, Kalita et al. 31 obtained superior results with the use of amitriptyline both for the outcome of 50% or more of reduction in pain intensity in the VAS reported by the participant (15 out of 46 [32.6%] versus 25 out of 47 [53.2%]; risk ratio: 0.61 [95%CI: 0.37–1.01]; *p* = 0.05) an for a reduction in the ODI greater than 20% (18 out of 46 [39.1%] versus 31 out of 47 [65.96%]; risk ratio: 0.59 [95%CI: 0.39–0.90]; *p* = 0.01). However, these results were classified as having a very low degree of evidence on the GRADE scale. Adverse events were reported in both groups, with no significant differences between them ( *p* = 0.48).

### Pregabalin compared to tramadol/acetaminophen

For the subgroup of patients without neuropathic pain, Sakai et al. 32 obtained significant pain improvement in the VAS at 4 weeks in both groups ( *p* < 0.05 for both) – very low degree of evidence (GRADE). However, in the tramadol/acetaminophen group, this improvement could already be observed after 2 weeks ( *p* < 0.05), that is, the effect in the pregabalin group took longer to be observed – very low degree of evidence (GRADE). As for the functional improvement measured by the RMDQ, no significant improvement was noted for the pregabalin group, whereas, for the tramadol/acetaminophen group, a significant improvement was observed after two weeks of administration – very low level of evidence (GRADE). Adverse events were reported in both groups, with a significantly higher number in the group that was using tramadol/acetaminophen (risk ratio: 0.59 [95%CI: 0.35–0.99]; *p* = 0.04).

### Pregabalin/celecoxib compared to monotherapy of each one

Comparing pregabalin and celecoxib in the subgroup of patients with unlikely neuropathic component, Romanò et al. 33 obtained a significant improvement in pain in the VAS only among patients using celecoxib (mean: 43.8 [standard deviation, SD: ± 12.9] to 32.5 [SD: ± 15.5]; *p* = 0.01) – very low degree of evidence (GRADE). When comparing the coadministration of pregabalin/celecoxib with pregabalin monotherapy in the subgroup of patients with an unlikely neuropathic component, Romanò et al. 33 obtained a greater decrease in pain with the combined use of drugs (mean: 45.1 [SD: ± 14.2] to 32.9 [ ±  13.9] versus 49.4 [SD: ± 13.2] to 50.7 [SD: ± 13.8]; *p* = 0.0002). Finally, in the comparison between the combned administration of pregabalin/celecoxib and celecoxib alone in the subgroup of patients with an unlikely neuropathic component, Romanò et al. 33 did not find superiority of the combined regimen compared to monotherapy in reducing pain (mean: 45.1 [SD: ± 14.2] to 32.9 [SD: ± 13.9] versus 43.8 [SD: ± 12.9] to 32.5 [SD: ± 15.5]; *p* = 0.9) – very low degree of evidence (GRADE). A total of 4 out of the 42 recruited patients discontinued the treatment due to adverse events (epigastralgia and/or nausea), with one taking pregabalin monotherapy, one taking celecoxib monotherapy, and two taking pregabalin plus celecoxib.

## DISCUSSION

The present review contains information from 5 studies totaling 242 participants. In the comparison between gabapentin and placebo, McCleane 29 reported a subtle reduction in the score on the pain scale only with the use of gabapentin, while Atkinson et al. 30 observed a decrease in pain in both groups, with no significant difference between them. Both studies demonstrated a greater presence of adverse events with the use of gabapentin than with placebo. Pregabalin, in turn, was compared with amitriptyline, tramadol/acetaminophen, celecoxib, pregabalin/celecoxib, and, finally, pregabalin/celecoxib was compared with celecoxib. In these comparisons, in no case was the pregabalin monotherapy superior to its comparator for pain relief, sometimes being inferior. 31 33 As for safety, there was no significant difference between the compared groups, with the exception of those in the study by Sakai et al., 32 in which patients using pregabalin reported fewer adverse events than those submitted to the coadministration of tramadol/acetaminophen.

### Overall completeness and applicability of the evidence

Only the study by McCleane 29 study entirely composed of patients of interest to the present review – individuals with nociceptive/mechanopostural CLBP, without radiculopathy or neuropathy –, which demonstrates the difficulty of finding studies with samples exclusively composed by this particular group. In the other studies, it was necessary to extract data from subgroups of the total set of patients, which significantly compromises the quality of the results for the purposes of the present review. Regarding our main outcomes, none of the studies reported the occurrence of any serious adverse event. Other outcomes, such as improvement in pain in the VAS and functional improvement by the ODI or RMDQ, were partially covered by the included articles; however, as highlighted, the difficulty in finding research entirely on patients of interest to us compromises the applicability of the results. Moreover, the fact that we chose not to examine the grey literature may have led to a higher risk of non-reporting bias (such as non-publication bias).

### Quality of the evidence

Although in general the studies included were not of very low methodological quality or high risk of bias, the quality of their evidence was greatly affected by several factors. The need to extract data from only a portion of the population, the very small sample size, and the risk of bias due to the non-blinding of the participants or the uncertainty regarding this process severely affected the quality of the evidence. This resulted in the fact that, finally, we maintained only two outcomes in one of the studies with moderate quality of evidence, with the other results being classified as of very low quality of evidence.

### Potential biases in the review process

We tried to avoid bias in the review process by conducting a comprehensive search without language restrictions, developing a comprehensive search strategy to identify all available evidence to answer our research question. However, a double full review by two reviewers was only performed after the initial exclusion of clearly ineligible articles by one of the authors. This represents a limitation, as it may increase the risk of human error in this selection. Furthermore, only one of the reviewers performed the data extraction and assessed the risk of bias of the included studies, which also represents a limitation.

Aiming to expand the scope of the primary search, allowing for the inclusion of studies with less objective definitions than the current ones for chronic pain, 34 35 36 we considered CLBP or back pain as pain for at least two months. While this may theoretically have limited the generalizability of our findings, 4 out of the 5 included studies defined CLBP as pain lasting longer than 12 weeks, and only 1 (McCleane 29 ) did not define it clearly. We have covered, in addition to publications on the subject, registered trial protocols. Furthermore, to ensure compliance with the revision primarily proposed, before starting the searches, we submitted our research project to the Research Project Management System (Sistema Gerenciador de Projeto de Pesquisa, SGPP, in Portuguese) of Hospital Israelita Albert Einstein, which was developed in accordance with the Lean Six Sigma requirements of the Process Improvement Program. Searches were not carried out in the grey literature, considering the generally lower methodological quality of these studies.

Finally, we endeavored to conduct a systematic review that followed the guidelines published and provided by the Cochrane Handbook for Systematic Reviews of Interventions. 28

### Agreements and disagreements with other studies or reviews

No study, except one 29 of those included in the present review, had a sample entirely composed of the population of interest to us, which limits the comparison with previous studies. However, our results were very similar to those found in another review 22 that evaluated the use of gabapentin and pregabalin for CLBP regardless of the neuropathic or radicular component. In its results, pregabalin was slightly less effective than other analgesics, such as amitriptyline, celecoxib, or tramadol/acetaminophen, and pregabalin used as adjuvant therapy (added to other medications – to celecoxib, in the case of the present review) did not show benefits ether. However, unlike our findings, the gabapentin group experienced no significant reduction in pain compared to the placebo group (mean difference = 0.22 units; 95%CI: -0.51–0.07; *p* = 0.14). In fact, one 31 of the studies we included did not support the use of gabapentin for a general sample of low back pain with and without pain radiating to the legs, but another, 29 only with patients without radicular pain or neuropathy, found a subtle pain reduction in the 0-10 verbal numeric rating scale during gabapentin use (of 7.10 to 6.39; *p* < 0.05), as well as an improvement in mobility (from 4.65 to 5.46; *p* < 0.01), with moderate quality of evidence (GRADE) for both results. Three main reasons may explain the differences in the findings of the other review: 29 1) the present review did not consider one of the studies included in this other review because the patients had associated leg pain; 2) we used only the population portion of the study by Atkinson et al. 30 with pain confined to the low back; and 3) the aforementioned review converted all study outcomes for pain relief expressed as continuous scores into a common 0-10 numerical rating scale.

In conclusion, the present review showed that there is still no quality information to support the use of pregabalin or gabapentin for the treatment of nociceptive/mechanopostural CLBP without radiculopathy or neuropathy, although the results suggest that the gabapentin may be a viable option. This corroborates the need for further data to fill the current gap in knowledge regarding this very relevant question.
