# Supplementary material for: Pregabalin and gabapentin for chronic low back pain without radiculopathy: a systematic review
Source: Arq Neuropsiquiatr. 2023 Jun 28;81(6):564–76. doi: 10.1055/s-0043-1764414 (PMC10306996; doi:10.1055/s-0043-1764414)
Supplement: Supplementary file 1 — Supplementary Material [file 10-1055-s-0043-1764414-s220039.pdf]

Supplemental File

Appendix 1 PubMed Search Strategy

Last search on August 20, 2022.

Using PubMed's MeSH (Medical Subject Heading) tool, the following search construction was performed:

- ((("Back Pain"[Mesh]) OR "Low Back Pain"[Mesh]) AND "Pregabalin"[Majr]) OR "Gabapentin"[Mesh].

The term "Pregabalin"[Majr] had the following inscription terms:

- (S)-3-(aminomethyl)-5-methylhexanoic acid
- 3-isobutyl GABA
- 3 isobutyl GABA
- GABA, 3-isobutyl
- 3-(aminomethyl)-5-methylhexanoic acid
- (R-)-3-isobutyl GABA
- (S +)-3-isobutyl GABA
- Lyrica
- CI 1008
- 1008, CI
- CI-1008
- CI1008

The filters used were:

- Controlled Clinical Trial
- Meta-Analysis
- Observational Study
- Randomized Controlled Trial
- Systematic Review

Through this search strategy, 755 records were found.

Appendix 2 Research strategy in the Cochrane Central Register of Controlled Trials (CENTRAL)

Last search on August 20, 2022.

Through the "Advanced Search" tool of the Cochrane Library, the "Search manager" was used to perform the following search construction:

Line		Search for	Hits
#1		(Back Pain) OR (Low Back Pain)	22956
#2		(Pregabalin) OR (Gabapentin)	4809
#3		#1 and #2	256

No filters were used.

Through this search strategy, 256 records were found.

Appendix 3 Research strategy at LILACS

Last search on August 20, 2022.

Through access to the VHL Regional Portal, the following search construction was performed:

- ((back pain) OR (low back pain)) AND ((pregabalin) OR (gabapentin)) AND (db:("LILACS"))

The only filter used was for the "LILACS" platform.

Through this search strategy, 8 records were found.

Appendix 4 Web of Science Search Strategy

Last search on August 20, 2022.

Through the advanced search tool of the Web of Science, the following search construction was performed:

Line		Search for	Hits
#1		TS = (Back Pain) OR TS = (Low Back Pain)	88825
#2		TS = (Pregabalin) OR TS = (Gabapentin)	13439
#3		#1 and #2	341

No filters were used.

Through this search strategy, 341 records were found.

Appendix 5 Embase Search Strategy

Last search on August 20, 2022.

Through access to Embase, the following search construction was performed:

('backache'/exp OR 'backache' OR 'back ache'/exp OR 'back ache' OR 'back pain'/exp OR 'back pain' OR 'back pain syndrome'/exp OR 'back pain syndrome' OR 'backpain'/exp OR 'backpain' OR 'dorsalgia'/exp OR 'dorsalgia' OR 'pain, back'/exp OR 'pain, back' OR 'low back pain'/exp OR 'back pain, low' OR 'chronic low back pain' OR 'loin pain' OR 'low backache' OR 'low backpain' OR 'lowback pain' OR 'lower back pain' OR 'lumbago' OR 'lumbal pain' OR 'lumbal syndrome' OR 'lumbalgesia' OR 'lumbalgia' OR 'lumbar pain' OR 'lumbar spine syndrome' OR 'lumbodynia' OR 'lumbosacral pain' OR 'lumbosacroiliac strain' OR 'pain, low back' OR 'pain, lumbosacral' OR 'strain, lumbosacroiliac') AND ('pregabalin'/exp OR '3 aminomethyl 5 methylhexanoic acid' OR '3 isobutyl 4 aminobutyric acid' OR '3 isobutyl gaba' OR '3 isobutylgaba' OR '4 amino 3

 © 2023. Academia Brasileira de Neurologia. All rights reserved.

isobutylbutyric acid' OR 'ci 1008' OR 'ci1008' OR 'lyrica' OR 'lyrica cr' OR 'pd 144723' OR 'pd144723' OR 'gabapentin'/exp OR '1 (aminomethyl) cyclohexaneacetic acid' OR 'ci 945' OR 'ci945' OR 'dineurin' OR 'gabalept' OR 'gabaliquid geriasan' OR 'gabatin' OR 'gantin' OR 'go 3450' OR 'go3450' OR 'goe 3450' OR 'goe3450' OR 'gralise' OR 'kaptin' OR 'keneil' OR 'neurontin' OR 'neurotonin' OR 'nupentin') AND [embase]/lim NOT ([embase]/lim AND [medline]/lim)

The only filter used was for "Sources": EMBASE (without MEDLINE).

Through this search strategy, 869 records were found.

Appendix 6 Criteria for assessing risk of bias for internal validity

To analyze the risk of bias of the study results, we used the Risk of Bias 2.0 tool (RoB 2). The effect of intention-to-treat (ITT) was of interest for this review, therefore assessments with RoB 2 were performed on this effect, and the results that we evaluated are those specified in tables 1 to 5 'Summary of findings'.

One of the reviewers (Rafael Trindade Tatit) assessed the risk of bias for each result. In case of doubts or open questions during the evaluation of the articles, a second reviewer (Carlos Augusto Cardim de Oliveira) was requested, reaching a consensus to reach a final decision. We evaluated the following types of bias, as described in Chapter 8 of the Cochrane Handbook for Systematic Reviews of Interventions Sterne JA, Savović J, Page MJ, Elbers RG, Blencowe NS, Boutron I, et al. RoB 2: a revised tool for assessing risk of bias in randomised trials. BMJ. 2019;366:l4898.

- Bias arising from the randomisation process
- Bias due to deviations from the intended interventions
- Bias due to missing outcome data
- Bias in measurement of the outcome
- Bias in selection of the reported result

For two studies (McCleane GJ. Gabapentin reduces chronic benign nociceptive pain: a double-blind, placebo-controlled cross-over study. PAIN Clin. 2000;12(2):81–5 and Romano CL, Romano D, Bonora C, Mineo G. Pregabalin, celecoxib, and their combination for treatment of chronic low-back pain. J Orthop Traumatol. 2009;10(4):185–91), as they were cross-over clinical trials, we planned to add an additional domain, the "Domain S", to assess the bias resulting from period effects, which they are systematic differences between the responses in the comparison groups not due to the interventions being compared, and the "carry-over" effect, an effect resulting from a previous situation or context. This has been done as recommended in Additional Considerations for Crossover Trials in RoB 2 (available in Risk of bias tools - RoB 2 for crossover trials), and in Chapter 23 of the Cochrane Handbook for Systematic Reviews of Interventions Higgins JPT, Thomas J, Chandler J, Cumpston M, Li T, Page MJ, Welch VA (editors). *Cochrane Handbook for Systematic Reviews of Interventions* version 6.2 [Internet]. Cochrane; 2021 [cited 2021 Dec 29]. Available from www.training.cochrane.org/handbook.

To deal with response bias, we used the signaling questions recommended in RoB 2 and a judgment was performed using the following options.

- 'Yes': if there is strong evidence that the question was answered in the study (ie, the study has a low or high risk of bias for the given direction of the question).
- 'Probably yes': a judgment was made that the question was answered in the study (ie, the study has a low or high risk of bias, given the direction of the question).
- 'No': if there is firm evidence that the question was not completed in the study (ie, the study has a low or high risk of bias for the given direction of the question).
- 'Probably no': a judgment was made that the question was not completed in the study (ie, the study has a low or high risk of bias due to the direction of the question).
- 'No information': if the study report does not provide sufficient information to make any judgment.

We use the algorithms proposed by RoB 2 to assign each domain one of the following levels of bias.

- 'Low risk of bias'
- 'Some concerns'
- 'High risk of bias'

We then perform an overall 'Risk of bias' rating for each pre-specified outcome in each study according to the following suggestions.

- 'Low risk of bias': we considered the trial to be at low risk of bias for all domains for this result.
- 'Some concerns': we judge that the clinical trial raises some questions in at least one domain for this outcome, but does not pose a high risk of bias for any domain.
- 'High risk of bias': we judge the clinical trial to have a high risk of bias in at least one domain for the outcome, or we judge the clinical trial to have some concerns for multiple domains in a way that substantially reduces confidence in the results.

We used the RoB 2 Excel tool to implement the RoB 2 (available in em Risk of bias tools - RoB 2 tool), adding the judgments for analysis of each study.

Appendix 7 Types of outcome measures

Primary outcomes:

- Reduction of pain intensity reported by the participant of 50% or more;
- Reduction of pain intensity reported by the participant of 30% or more;
- Assessment of functional improvement (Oswestry Disability Index or Roland Morris Disability Questionnaire);

 © 2023. Academia Brasileira de Neurologia. All rights reserved.

- Occurrence of a serious adverse event, including any untoward medical occurrence or effect that, at any dose, results in: death, life-threatening, hospitalization or prolongation of existing hospitalization, disability (persistent or significant), whether a congenital anomaly or birth defect, or a major medical event that could compromise the patient or may require intervention to avoid one of the above characteristics or consequences.

Secondary outcomes:

- Patient Global Impression of Change - PGIC; “much or very much improved”;
- Any pain-related outcome indicating some improvement;
- Adverse events such as drowsiness and dizziness;
- Other adverse events in addition to those mentioned above.

 © 2023. Academia Brasileira de Neurologia. All rights reserved.
